# An audit of food and beverage advertising on the Sydney metropolitan train network: regulation and policy implications

**DOI:** 10.1186/s12889-017-4433-2

**Published:** 2017-05-22

**Authors:** Emma Sainsbury, Stephen Colagiuri, Roger Magnusson

**Affiliations:** 1 The Boden Institute of Obesity, Nutrition, Exercise & Eating Disorders, Charles Perkins Centre, The University of Sydney, Sydney, NSW 2006 Australia; 2 Sydney Law School, The University of Sydney, Sydney, NSW 2006 Australia

**Keywords:** Australia, Advertisements, Food, Beverage, Train stations, Unhealthy, Regulation

## Abstract

**Background:**

Increased marketing of energy-dense, nutrient-poor foods has been identified as a driver of the global obesity epidemic and a priority area for preventative efforts. Local and international research has focused on the unhealthiness of television advertising, with limited research into the growing outdoor advertising industry. This study aimed to examine the extent of food and beverage advertising on the Sydney metropolitan train network, and to assess the nutritional quality of advertised products against the Australian Guide to Healthy Eating.

**Methods:**

All 178 train stations on the Sydney metropolitan train network were surveyed in summer and winter. A survey tool was developed to collect information for all advertisements on and immediately surrounding the train station. Information included product, brand, location and advertisement format. Advertisements were coded by nutrition category, product subcategory and size. Chi-square, ANOVA and ANCOVA tests were conducted to test for differences in the amount of food and beverage advertising by season and area socioeconomic status (SES).

**Results:**

Of 6931 advertisements identified, 1915 (27.6%) were promoting a food or beverage. The majority of food and beverage advertisements were for unhealthy products; 84.3% were classified as discretionary, 8.0% core and 7.6% miscellaneous. Snack foods and sugar-sweetened beverages were the most frequently advertised products, regardless of season. Coca-Cola and PepsiCo were the largest advertisers on the network, contributing 10.9% and 6.5% of total advertisements respectively. There was no difference in the mean number of food and beverage advertisements by area SES, but the proportion of advertising that was for discretionary foods was highest in low SES areas (41.9%, *p* < 0.001).

**Conclusions:**

The results indicate that, irrespective of season, food and beverage advertisements across the Sydney metropolitan train network are overwhelmingly for unhealthy (discretionary) products. The results of this study highlight the inadequacy of Australia’s voluntary self-regulatory system in protecting members of the public from exposure to unhealthy food advertising. Regulatory action by government, such as placing a cap on the amount of unhealthy food advertisements, or requiring a proportion of all advertising to be for the promotion of healthy foods, is required to address this issue.

**Electronic supplementary material:**

The online version of this article (doi:10.1186/s12889-017-4433-2) contains supplementary material, which is available to authorized users.

## Background

Overweight and obesity are a major public health issue, responsible for 5.5% of Australia’s burden of disease [1]. In 2014–2015, 63% of adults and 27% of children were classified as overweight or obese [2]. There are variations in the distribution of overweight and obesity across Australia, with lower socioeconomic status (SES) groups disproportionately more likely to be above a healthy weight [3]. Overweight and obesity are estimated to cost Australia $56.6 billion annually [4] and are responsible for 52% of the burden of disease from diabetes, 23% of the coronary heart disease burden, and 17% of the stroke burden [1]. The causes of overweight and obesity are multifactorial but changes in the food environment, including the increased marketing of energy-dense nutrient-poor foods, have been identified by the World Health Organization (WHO) as a driver of the global obesity epidemic and a priority area for preventative efforts [5–8]. There is substantial evidence showing the influence of advertising on the purchasing, consumption and taste preferences of children [8–10], and evidence is growing for the effects of food advertising on the consumption behaviours of adults [11–13]. In 2010, the WHO passed a resolution urging Member States to take measures to reduce children’s exposure to, and the power of, marketing of foods high in saturated fats, trans-fatty acids, sugar and/or salt [6, 14]. In 2016, the Commission on Ending Childhood Obesity noted with concern the failure of Member States to implement this resolution [15].

The food industry is increasingly utilising non-broadcast channels such as out-of-home or outdoor advertising for product marketing, as seen in the growing annual revenue of this industry [16]. In Australia, outdoor advertising reaches 12.2 million people daily, with an average of 26 advertisements viewed daily per person [17, 18]. The Sydney Train network is one prominent setting for advertising, with one million passenger journeys recorded per weekday [19]. Demographic profiling of train commuters revealed close to 45% are young adults aged 18–34 years, with children and adolescents making up approximately 15% of all patrons [20]. A 2013 study revealed 67% of people noticed advertising at train stations more than other places, and 21% of station time is directly spent viewing cross-track advertisements [21]. Stations within the Sydney Central Business District (CBD) and surrounding suburbs are larger, record the highest number of commuter journeys per year [22], and have more space available for advertising, making them particularly influential settings.

International studies of outdoor advertising in the United States [23, 24], United Kingdom [25] and New Zealand [26] have reported a large amount of advertising for unhealthy foods and beverages. While Yancey et al. reported a higher density of unhealthy food advertising in low-income, racial/ethnic minority areas [24], the literature is conflicting; as studies by Maher et al. and Adams et al. have reported more unhealthy food advertising in higher SES areas [25, 26]. Australian research has focused on television food advertising [27–30], with only a small body of literature investigating the extent of outdoor food and beverage advertising. Kelly and colleagues investigated food and beverage advertising surrounding primary schools in New South Wales (NSW) and identified train stations as the setting with the highest proportion of non-core food advertising (90%) [31]. Recent studies investigating advertising at Melbourne [32], Sydney [33] and Perth [34] transit stops reported a high proportion of advertising for snack foods, sugar-sweetened and intense-sweetened beverages, and alcohol. Settle and colleagues [32] found that 30% of sampled transit stops in Melbourne displayed food advertisements, with some variation between the types of food and beverages advertised according to the level of socioeconomic disadvantage of the suburb. No study has provided a complete audit of food advertising across the train network of a major Australian city, assessing the nutritional quality of advertised foods and considering seasonal effects on advertising. The company and brand information of advertised products have not been reported in any existing study, despite literature showing the direct effects of repeated brand exposure on preference and desire for products [9, 35]. In addition, few studies of outdoor food advertising have explored the policy implications of their findings and made recommendations for regulatory change.

### Regulation of food and beverage advertising in Australia

In Australia, outdoor food advertising is ostensibly governed by a number of mandatory and voluntary standards. The former include the Australian Consumer Law [36], which includes legal restrictions on engaging in misleading or deceptive conduct. The Australian Consumer Law is administered by the Australian Competition and Consumer Commission (ACCC), to which complaints about breaches of the law may be directed. The peak body for advertising self-regulation, the Advertising Standards Bureau (ASB) oversees a number of voluntary codes created by the food and advertising industries. These include the Australian Association of National Advertisers (AANA) Code of Ethics [37], the AANA Food & Beverages Advertising & Marketing Communications Code [38], and the AANA Code of Advertising & Marketing Communications to Children [39]. Complaints about breaches of codes administered by the ASB can be directed to the Advertising Standards Board, whose members are independent of industry and are intended to represent the diversity of Australian society. However, these codes do not impose controls on the nutritional quality of foods advertised in outdoor settings. The Australian Food and Grocery Council, which represents manufacturers of food, beverage and grocery brands in Australia, also manages two voluntary initiatives that relate to food advertising to children: the Responsible Children’s Marketing Initiative (RCMI) [40] and the Quick Service Restaurant Initiative for Responsible Advertising and Marketing to Children (QSRI) [41]. Neither initiative imposes meaningful constraints on the foods that can be advertised on the public transport system. For example, signatories to the RCMI agree that advertising should represent “healthier dietary choices, consistent with established scientific or Australian government standards” [40]. However, companies are permitted to establish their own criteria for determining what foods represent healthier dietary choices, and the obligations in the RCMI only apply to advertising and marketing which is directed primarily to children under 12 years. Advertising in media where children comprise less than 35% of the audience are excluded from both the RCMI and QSRI.

A separate industry body, the Outdoor Media Association (OMA), administers a Code of Ethics [42] that requires members to adhere to the AANA codes mentioned above, as well as the Alcohol Beverages Advertising Code (ABAC) [43]. ABAC is administered by a 6-member management committee with majority representation coming from the industry associations for beer, wine, and spirits as well as the peak body representing the Australian advertising industry. The ABAC sets standards around the responsible and moderate portrayal of alcohol consumption, but does not restrict the volume or location of alcohol advertisements [43]. The OMA’s voluntary Alcohol Advertising Guidelines provide that alcohol advertisements should not be located within a 150 m sight line of a school; however, this guideline does not apply if the school is located in the vicinity of a pub, bottle shop or venue selling alcohol [44]. In summary, although the regulatory environment of food and beverage advertising in Australia is ostensibly complex, with a number of voluntary standards providing guidance to the food and beverage industries, in reality they impose few if any meaningful constraints on the nutritional content of food advertising, and on the placement of food and alcoholic beverage advertisements.

Public health organisations have expressed concern over the inadequacy of the voluntary self-regulatory system in Australia, advocating for the introduction of government restrictions on unhealthy food advertising [45, 46]. Although the case for reform is usually framed in terms of protecting children from exposure to unhealthy food advertising, evidence also supports the link between advertising and consumption of unhealthy food by adults [11–13]. In 2011 the Commonwealth House of Representatives Standing Committee conducted an inquiry into the regulation of billboard and outdoor advertising to ensure the content of advertisements met consumer standards [47]. Recommendations were made for a specific code of practice for outdoor advertising, and for regular review of advertisements by both the ASB and the Government [47]. The influence of these recommendations on the outdoor advertising environment is yet to be reported.

This study aimed to examine the extent of food and beverage advertising on the Sydney metropolitan train network, and to assess the nutritional quality of advertised products against the Australian Guide to Healthy Eating in order to answer the following research questions: 1) What is the level of public exposure to unhealthy food and beverage advertising on Sydney metropolitan train stations? 2) Does the amount and type of food and beverage advertising vary by area socioeconomic status? The findings of this study will help to inform future policy recommendations to reduce exposure to unhealthy food advertising in outdoor settings.

## Methods

### Sampling

All 178 train stations on the Sydney metropolitan network were surveyed for advertisements. Train stations were stratified by SES using the Australian Bureau of Statistics 2011 Index of Relative Socioeconomic Advantage and Disadvantage by postal area [48]. Stations were grouped as low (<1000), medium (1000–1100) and high (>1100) SES, based on previous studies [31, 33].

### Data collection

Data were collected over 1 week in February (summer) and 1 week in July (winter) 2016. The two time points were included to test for seasonal variation in advertisements. Daily permits were purchased from Sydney Trains to allow station access and permission to photograph. Each train station was scanned by one Masters of Nutrition student from the University of Sydney. For Central Station, only platforms 16–25 (suburban lines) and the surrounding concourse were included in the survey.

A survey tool was developed to record information on all advertisements, including non-food advertisements, and photographs were taken to assist with any ambiguity surrounding coding. Development of the survey tool was guided by optimal methods of measurement proposed by Kelly et al. [49]. Pilot data collection was completed on 60 train stations in November 2015 to test the feasibility of the study protocol and survey tools. Student training sessions were held at Central station prior to each data collection period to ensure consistency and accuracy in scanning measures and documentation of advertisements. Surveys completed during training were checked against a reference copy completed by the first author, and any discrepancies were addressed with the students prior to commencing the study. An advertisement was defined as any commercial billboard or poster, temporary flyers, branded furniture, vending machines, and experiential displays promoting a product, service or brand. Public service announcements from Sydney Trains (e.g. ticketing and event information), business signage, advertisements within surrounding shops and venues, advertisements smaller than A4 size, and historic advertisements preserved as artwork at Museum station were excluded. Information collected included: 1) Product name and description; 2) Product brand/company; 3) Advertisement location; 4) Advertisement format [see Additional file 1]. Other relevant information, including any nutrition information on the advertisement, was also recorded. Advertisement locations included the train station concourse, station platform, cross-track billboards, or any external advertisements designed to be seen by commuters standing on the platform or entering/exiting the station. Advertisement formats included print, digital, video, sampling and experiential, and exterior surfaces of vending machines. For rotating billboards, all advertisements shown within one complete rotation were recorded. Coding queries were discussed within the project team to reach agreement.

### Advertisement coding

All advertisements were coded by the first author (ES), a research dietitian. Advertisements were classified as food or non-food. A food-based coding system previously used to assess television marketing to children [30, 31] was used to categorise food and beverage advertisements as core (healthy foods and beverages recommended for daily consumption), discretionary (high fat, sugar and/or salt foods and beverages not recommended for daily consumption), or miscellaneous (tea and coffee, nutritional supplements, and brand-only advertisements) based on the Australian Guide to Healthy Eating [50]. These groups were further divided into 32 product subcategories. An additional coding category was included for intense-sweetened beverages, adopting the definition used in the Australian Health Survey conducted by the Australian Bureau of Statistics: ‘cordials, soft drinks and flavoured mineral waters, and electrolyte and energy drinks that have been artificially sweetened’ [51]. For the purposes of this study, full-fat and reduced-fat flavoured milks were coded as a sugar-sweetened beverage. A further subcategory for food-related health campaigns was also included. Advertisements promoting multiple products were coded based on the major product shown. For vending machines promoting a different product on each panel, each advertisement was coded separately. Using the photographs and information on format and location, advertisements were also coded into three size categories modified from Kelly et al. [31]: small (> 21 × 30 cm and <0.9 × 1.4 m; temporary posters, flyers and banners approximately A4 size); medium (> 0.9 × 1.4 m and <2.0 × 2.5 m; size of billboards on concourse and platform); large (> 2.0 × 2.5 m; size of cross-track billboards).

### Data analysis

Data were analysed using IBM SPSS Statistics for Windows Version 22.0 [52]. Descriptive statistics, including the number and proportion of food and beverage advertisements overall, and by nutrition category, product sub-category and company were calculated for each season. Pearson chi-square goodness of fit tests were applied to test for equal proportions of total, core, discretionary and miscellaneous food advertising between seasons (Table 1). We considered results significant at the 5% level. To enable standardized comparisons between low, medium and high SES areas, the mean number and standard deviation (SD) of advertisements per station was calculated for each group. One-way analysis of variance (ANOVA) tests were conducted to compare the means for total food and beverage advertising, and for each nutrition category (Table 3). For models that resulted in *p* < 0.05, post-hoc Tukey tests were conducted for pairwise comparisons. An online distance calculator was used to measure the direct line distance in kilometres (km) of each train station from the Sydney CBD. Given the closer proximity of high SES stations to the Sydney CBD, this was adjusted for in analysis. One-way between-groups analysis of covariance (ANCOVA) tests were conducted to compare the estimated marginal means for each SES group, adjusting for distance to the CBD and total number of advertisements (Table 3).

**Table 1:** Number and proportion of food and beverage advertisements by nutrition category, for summer and winter

	Summer	Winter	Total	*p*-value
Total number of advertisements	3657	3274	6931	
Number (%) of food and beverage advertisements	999 (27.3)	916 (28.0)	1915 (27.6)	0.06
Mean number (SD) of food and beverage advertisements per station	5.6 (11.6)	5.2 (11.0)	10.8 (20.2)	
Median (min-max)	2.0 (0–95)	2.5 (0–122)	5.0 (0–152)	
Number (% of total food) of core food advertisements	64 (6.4)	90 (9.8)	154 (8.0)	0.04
Mean number (SD) of core food advertisements per station	0.4 (0.8)	0.5 (1.2)	0.9 (1.8)	
Median (min-max)	0.0 (0–4)	0.0 (0–8)	0.0 (0–10)	
Number (% of total food) of discretionary food advertisements	859 (86.0)	756 (82.5)	1615 (84.3)	0.01
Mean number (SD) of discretionary food advertisements per station	4.8 (10.2)	4.3 (9.9)	9.1 (17.9)	
Median (min-max)	2.0 (0–91)	2.0 (0–114)	4.0 (0–142)	
Number (% of total food) of miscellaneous food advertisements	76 (7.6)	70 (7.6)	146 (7.6)	0.62
Mean number (SD) of miscellaneous food advertisements per station	0.4 (1.6)	0.4 (1.7)	0.8 (2.6)	
Median (min-max)	0.0 (0–11)	0.0 (0–16)	0.0 (0–24)	

*p*-value for test of equal proportions between summer and winter

## Results

A total of 6931 advertisements were observed across the 178 train stations, of which 27.6% (1915/6931) were promoting a food or beverage. There was a small difference in the proportion of food and beverage advertisements between seasons but this was not statistically significant (χ^2^_1_ = 3.60, *p* = 0.06) (Table 1). Over half of all food and beverage advertisements were located on the station platform (53.6%) or were visible from the platform as a cross-track advertisement (6.2%). The remaining advertisements were located on the station concourse (34.6%) or immediately external to the station (5.6%). Just over 50% of all food advertisements were on vending machines. The majority of food and beverage advertisements across the network were medium sized (80.8%), with 12.1% classified as small and 7.2% as large-sized advertisements.

**Fig. 1:**
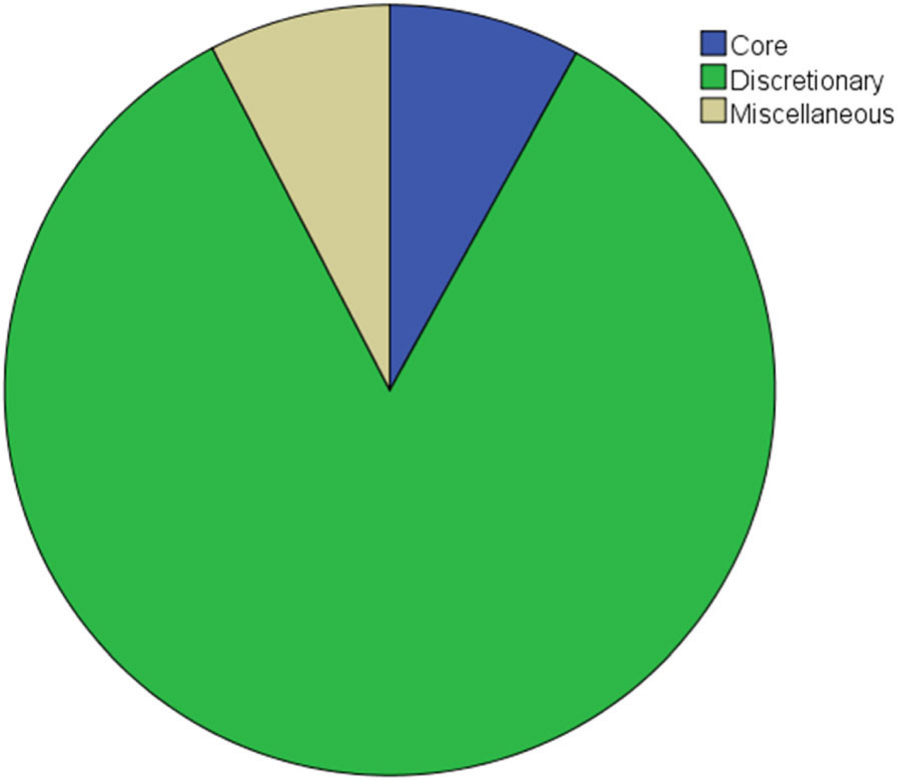
Breakdown of food and beverage advertisements by nutrition category

Of the food and beverage advertisements, there were a significantly greater proportion of advertisements for discretionary foods than for core foods (84.3% vs. 8.0%) (Fig. 1). There was a significant relationship between the nutritional quality of advertisements and the season; chi-square goodness of fit tests revealed more discretionary food advertisements in summer (*p* = 0.01) and more core food advertisements in winter (*p* = 0.04) (Table 1). This variation was primarily due to a higher proportion of vegetable soup and bottled water advertisements in winter, and more advertisements for biscuits in summer (Table 2). Within the core food category, the majority of advertisements were either bottled water vending machines (74.4%), or billboard advertisements for bottled water (11%). Excluding bottled water advertisements from analysis, only 1.3% of all food and beverage advertising was for core foods.

**Table 2:** Contribution of product subcategories to total food and beverage advertising on the Sydney metropolitan train network

Advertisement subcategory	Number of (% of total food) advertisements		Total (%)
	Summer	Winter	
Core			
Bottled water (including mineral and soda water)	59 (5.9)	72 (7.9)	6.8
Soups, salads and sandwiches, including frozen meals (<10 g fat/serve), soups (<2 g fat/100 g, excludes dehydrated), sandwiches, mixed salads and low fat savoury sauces (<10 g fat/100 g; includes pasta simmer sauces), low fat mixed meals or side dishes (<10 g fat)	0 (0)	15 (1.6)	0.8
Breads (include high fibre, low fat crackers), rice, pasta and noodles	4 (0.4)	0 (0)	0.2
Vegetables and vegetable products	0 (0)	3 (0.3)	0.2
Low fat/reduced fat milk, yoghurt, custard (<3 g/100 g fat) and cheese (<15 g fat/100 g); includes 50% reduced fat cheddar, ricotta and cottage and their alternatives (e.g. soy) (including probiotic drinks)	1 (0.1)	0 (0)	0.1
Discretionary			
Snack foods, including chips, savoury crisps, extruded snacks, popcorn, sugar-sweetened fruit and vegetable products, and sugar coated and salted nuts	253 (25.3)	226 (24.7)	25.0
Sugar sweetened drinks including soft drinks, cordials, electrolyte drinks and flavour additions (e.g. Milo)	230 (23)	210 (22.9)	23.0
Intense-sweetened beverages	191 (19.1)	167 (18.2)	18.7
Alcohol	52 (5.2)	67 (7.3)	6.2
High fat savoury biscuits, sweet biscuits, cakes, muffins, pastries, pies	64 (6.4)	9 (1.0)	3.8
Fast food restaurants/meals (including pizza, burgers, ‘healthy’ alternatives from fast food restaurants)	25 (2.5)	24 (2.6)	2.6
Chocolate and confectionary (including regular and sugar-free chewing gum)	21 (2.1)	26 (2.8)	2.6
Ice cream and iced confection	10 (1.0)	16 (1.7)	1.4
High fat/sugar/salt spreads (including yeast extracts, excludes peanut butter), oils, high fat savoury sauces, mixed meals and side dishes (>10 g fat/100 g), meal helpers (including stocks, tomato paste) and soups (>2 g fat/100 g, tinned and all dehydrated)	12 (1.2)	7 (0.8)	1.0
Fruit juice and fruit drinks	0 (0)	1 (0.1)	0.1
Full cream milk, yoghurt, custard, dairy desserts (>3 g fat/100 g) and cheese (25% reduced fat and full fat varieties) and high salt cheese (including haloumi and feta) and their alternatives	1 (0.1)	0 (0)	0.1
Miscellaneous			
Brand only – nil specific products mentioned	71 (7.1)	49 (5.3)	6.3
Supermarkets - non-specified (generic supermarket ads or not clearly for core or discretionary)	3 (0.3)	11 (1.2)	0.7
Vitamin and mineral supplements	1 (0.1)	2 (0.2)	0.2
Tea and coffee	0 (0)	3 (0.3)	0.2
Supermarkets – advertising core foods	0 (0)	4 (0.4)	0.2
Supermarkets – advertising discretionary foods	0 (0)	1 (0.1)	0.1

Table 2 shows the breakdown of advertisements by product subcategory. Snack foods, in particular potato chips, were the most frequently advertised product (25.0%), followed by sugar-sweetened beverages (23.0%) and intense-sweetened beverages (18.7%). Within the sugar-sweetened beverage subcategory, the majority of advertisements were for flavoured milks (53.0%) and soft drinks (35.9%), with sports drinks, cordials, energy drinks, iced teas, and flavoured mineral waters each contributing less than 4%. Advertisements for alcohol contributed 6.2% of food and beverage advertising, and 1.7% of total advertising. An additional two advertisements made reference to alcohol, despite promoting a non-alcoholic product.

**Fig. 2:**
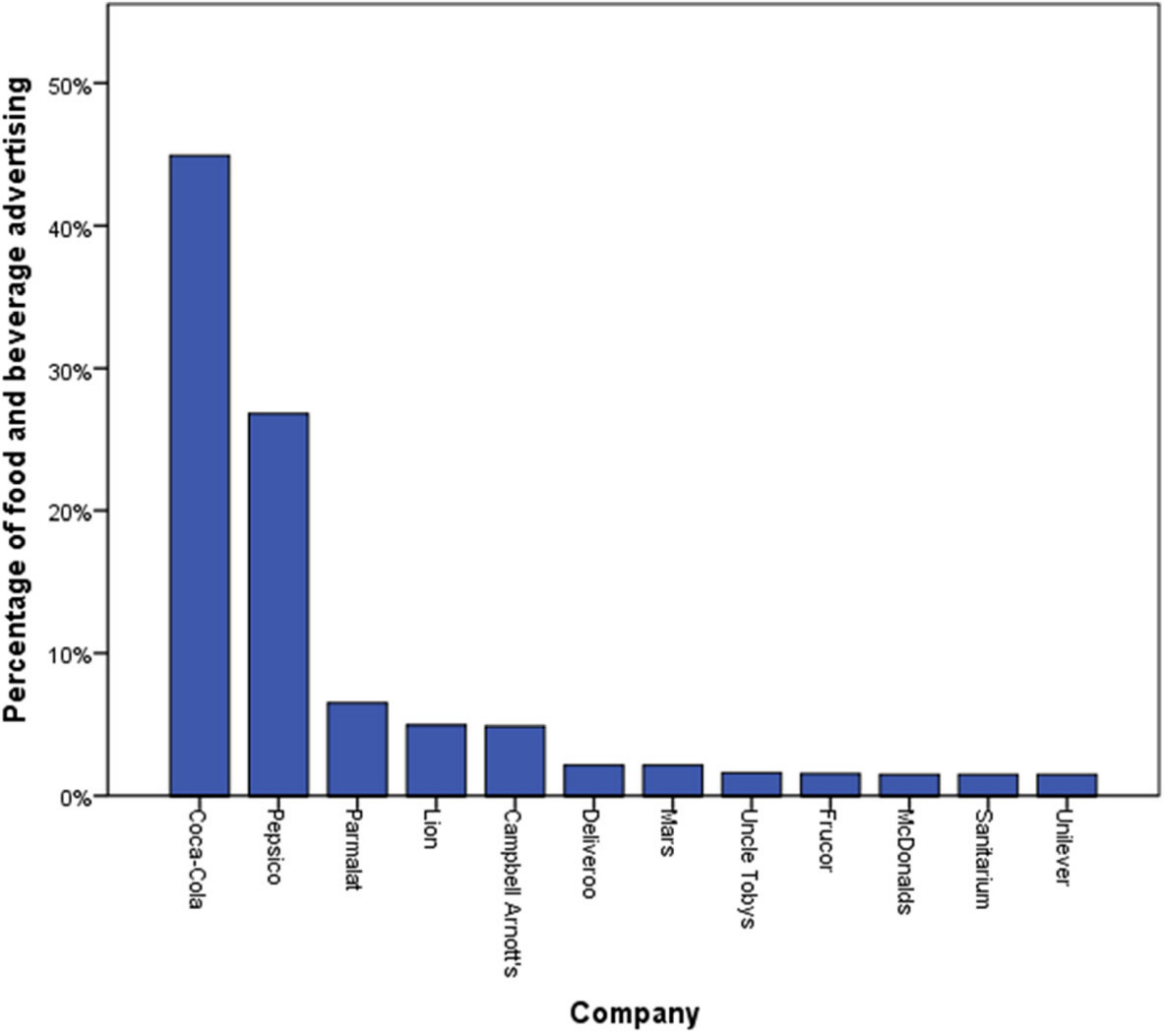
Companies with the highest proportion of food and beverage advertising on the Sydney metropolitan train network

Figure 2 shows the companies that contributed the greatest amount of food and beverage advertising on the train network. Of the advertisements by Coca-Cola, 46.4% were for intense-sweetened soft drinks, 22.8% were for sugar-sweetened soft drinks, 17.4% for bottled water, 11.3% for flavoured milk, and 2.1% were brand-only. Despite food advertisements comprising just over a quarter of all advertising on the network, Coca-Cola and PepsiCo (which encompasses PepsiCo beverages and The Smith’s Snackfood Company) remained the largest advertising companies overall, contributing 10.9% and 6.5% of total advertisements respectively.

While there were a greater number of food and beverage advertisements in medium and high SES areas, the difference between groups was not statistically significant [*F* (2, 175) = 0.53, *p* = 0.59]. There was no significant difference in the number of core and discretionary advertisements between SES groups, but post-hoc Tukey tests revealed a slightly higher number of miscellaneous food advertisements at stations in medium SES areas compared with low SES areas (*p* = 0.04) (Table 3). After adjusting for both distance to the CBD and total number of advertisements, there was no significant difference in the amount or type of food and beverage advertising between groups. A chi-square goodness of fit test was conducted to test for equal proportions of discretionary food advertising in low, medium and high SES areas. The results were significant, revealing low SES areas had a higher proportion of total advertisements that were for discretionary foods and beverages (41.9%; χ^2^_2_ = 229.32, *p* < 0.001).

**Table 3:** Comparison of the mean number of food and beverage advertisements per station, according to socioeconomic status

	low SES	medium SES	high SES	*p*-value
	(*n* = 65)	(*n* = 89)	(*n* = 24)	
Mean distance from the CBD (km)	23.9	16.0	13.3	
Range (km)	3.9–51.4	0.2–51.9	2.9–28.1	
Total number of advertisements	1196	4855	880	
Mean number (SD) of advertisements per station	18.4 (42.4)	54.6 (129.7)	36.7 (57.0)	0.02
Number (%) of food and beverage advertisements	568 (47.5)	1078 (15.6)	269 (30.6)	
Mean number (SD) of food and beverage advertisements per station	8.7 (19.7)	12.1 (22.4)	11.2 (11.7)	0.59
Estimated marginal mean (SE)	12.1 (1.3)	9.4 (1.1)	11.9 (2.1)	0.24
Number (%) of core food advertisements	55 (4.6)	75 (1.5)	24 (2.7)	
Mean number (SD) of core food advertisements per station	0.9 (1.9)	0.8 (1.9)	1.0 (1.3)	0.93
Estimated marginal mean (SE)	1.1 (0.2)	0.7 (0.2)	1.0 (0.3)	0.15
Number (%) of discretionary food advertisements	501 (41.9)	892 (18.4)	222 (25.2)	
Mean number (SD) of discretionary food advertisements per station	7.7 (18.2)	10.0 (19.4)	9.3 (10.2)	0.73
Estimated marginal mean (SE)	10.5 (1.3)	7.8 (1.1)	10.0 (2.1)	0.26
Number (%) of miscellaneous food advertisements	12 (1.0)	111 (2.3)	23 (2.6)	
Mean number (SD) of miscellaneous food advertisements per station	0.2 (0.8)	1.3 (3.5)	1.0 (1.9)	0.02
Estimated marginal mean (SE)	0.6 (0.3)	1.0 (0.2)	0.9 (0.4)	0.57

*n* values indicate number of train stations. *p*-value for comparison of means between low, medium and high SES areas

## Discussion

This study aimed to determine the level of public exposure to unhealthy food and beverage advertising across the entire Sydney metropolitan train network. The results reveal a high proportion of unhealthy (discretionary) food and beverage advertising on Sydney train stations, with close to 1 in 4 advertisements promoting energy-dense, nutrient-poor foods that conflict with the Australian Dietary Guidelines (Table 1). Of added concern was the small proportion of healthy (core) food advertising (8% of total food and beverage advertising), when compared with television. For example, one international study that surveyed children’s television advertising across 13 countries reported 22% of all advertising to be for core foods [53]. Furthermore, in our study, advertisements for bottled water on the exterior surface of vending machines made up the majority of core food advertising. These advertisements are unlikely to translate into healthy food choices, as the vending machines were primarily stocked with chocolate and confectionary, snack foods and sugar- sweetened beverages.

Regardless of season, snack foods, sugar-sweetened beverages and intense-sweetened beverages were the most heavily advertised product categories. Settle et al. reported a similar product breakdown in their audit of Melbourne public transit stops, supporting the transferability of our results to the broader Australian context [32]. The lower volume of advertisements for water-based sugar-sweetened beverages appears promising, given the evidence identifying high intakes of soft-drinks as a probable causal factor in weight gain [5]. However, soft-drink company Coca-Cola was the most frequently marketed brand across the network, the likely effects of which are to build familiarity and preference for Coca-Cola products [9, 35].

Approximately 60 advertisements for alcohol were recorded across the network per time point, a lower proportion than recorded in other studies of outdoor advertising [31, 34]. This is likely a conservative estimate of alcohol advertising exposure, as advertisements associated with surrounding liquor stores and licensed venues were excluded for the purposes of this study. Some alcohol companies also extended their brands onto non-alcoholic products such as soft-drinks and while not coded in the alcohol category, these advertisements are still considered an alcohol brand extension under the self-regulatory Alcohol Beverages Advertising (and Packaging) Code (ABAC) [43]. The AANA Code for Marketing and Advertising Communications to Children states that advertising to children must not be for, or relate to, alcohol products or companies. Whether deliberately targeting children or not, alcohol advertisements are continuing to reach a substantial underage audience, with close to 15% of all Sydney train users being children and adolescents [20]*.* Systematic reviews of longitudinal studies have shown a link between exposure to alcohol advertisements and both initiation and frequency of drinking [54, 55], creating a strong case for the restriction of alcohol advertising in public areas such as train stations.

In its set of recommendations on the marketing of foods and non-alcoholic beverages to children, WHO emphasized that the goal of policy should be to reduce both exposure to, and the persuasive power of foods high in saturated fats, trans-fatty acids, free sugars, or salt [6]. It was beyond the scope of this paper to examine the persuasive marketing techniques used by food companies, but the intentional placement of advertisements both across the network and at the station level suggest public exposure to these advertisements is high. Over 50% of food and beverage advertisements were visible from the station platform, an area of the station with the highest commuter dwell time [21]. Further, close to 90% of all food and beverage advertisements were displayed on medium or large sized billboards which are more likely to be noticed by commuters.

The second question addressed by this study is whether the amount and type of food and beverage advertising on Sydney metropolitan train stations varies by area SES. The results indicate no significant difference in the number of food and beverage advertisements between stations in low, medium and high SES areas, even after adjusting for distance to the CBD. However, the proportion of total advertisements that were for foods and beverages, in particular unhealthy foods and beverages, was significantly higher in low SES areas (41.9%, *p* < 0.001), in line with previous work by Kelly et al. [31]. These results are likely due to the fact that many of the stations in low SES areas were single platform stops with vending machine advertising only. While train commuters are repeatedly exposed to unhealthy food advertising in all SES areas, we hypothesise that the visibility and impact of unhealthy food advertising may be greater at stations in low SES areas as it comprises a greater proportion of total advertising. Australian cross-sectional research has shown an association between low SES and poor dietary intake [56, 57], highlighting the need for advertising regulation that can correct rather than exacerbate the health inequalities experienced by more disadvantaged communities.

### Limitations

The coding system used for this study classified items by general food groups, rather than applying nutrient criteria to products. In the absence of a detailed nutritional assessment of all foods shown in advertisements, this study may have coded foods that, while discretionary under the Australian Guide to Healthy Eating, do hold some nutritional value.

The replacement of traditional print advertising with digital and video billboards at some stations has greatly increased both the volume of advertisements, and rate of advertisement turnover. The introduction of dynamic time-sharing whereby companies can purchase digital advertising space by the hour means two data collection points may not provide an accurate picture of advertising across the network.

### Implications for policy

The voluntary self-regulatory initiatives that ostensibly regulate food and beverage advertising in Australia impose few, if any, meaningful constraints on the advertising of food and beverages (including alcohol) on the public transport system. As a result, both adults and children are repeatedly exposed to advertisements for unhealthy foods and beverages. The Australian Government’s Best Practice Regulation Handbook states that self-regulation is an appropriate option if ‘there is no strong public interest concern, in particular any major public health and safety concerns’ [58]. Given the proven association between advertising exposure and unhealthy dietary behaviours [8–13, 59], the results of this study support the case for government to set ground rules for outdoor advertising in order to substantially shift food advertising on public transport away from unhealthy towards healthy foods and beverages.

A number of regulatory approaches could be implemented to address this issue, the most restrictive being a total ban on unhealthy food and beverage advertising across the Sydney train network. Sydney Trains generated over $12 million in advertising revenue in the 2013–14 financial year, and is anticipated to generate at least $100 million over the subsequent 5 years [60, 61], a significant proportion of which (up to $28 million, based on our study) would be expected to come from food and beverage advertising. This regulatory approach is therefore likely to be met by opposition due to its impact on revenues. An alternative approach would be to set interim and final targets for reductions in the overall volume of unhealthy food and beverage advertising that are phased in over time. While targets could aim to reduce the total number, size or proportion of unhealthy food advertisements, implementing targets for the proportion of advertising allocated to unhealthy foods and beverages at each station may be a favourable strategy, given the greater impact it is expected to have in low SES communities.

Healthy food advertising and health campaigns have been shown to have a small but significant effect on increasing healthy food choices and reducing caloric intake, even when shown in combination with unhealthy food advertising [62]. Another potential regulatory option would be to significantly increase the proportion of train station advertising allocated to the promotion of healthy, core foods and beverages. In order to encourage a higher proportion of core foods and beverages, the NSW Government could potentially use higher pricing strategies for unhealthy food advertisements, provided it remained accountable for meeting interim targets for reductions in unhealthy food advertising across the network. Contractual agreements between the NSW Government and the private advertising companies that manage Sydney train station advertising would need to be amended to reflect these actions [63]. While allocating a share of billboard advertising to healthy food promotion provides one potential solution, focusing on billboard advertising alone is insufficient. This study highlights the significant contribution of vending machines to total food advertising, and the need for regulation that either limits the number of machines, or permits only unbranded machines on train station property.

Currently in Australia there is no nutrient profiling system for classifying food advertisements as healthy or unhealthy. Companies that have signed on to the RCMI and QSRI – the food industry pledges that aim to moderate unhealthy food advertising to children – are therefore permitted to establish their own criteria [40, 41]. A nutrient profiling model is necessary to ensure consistency in the foods and beverages that are restricted from being advertised, such as that used in the United Kingdom [64]. Alternatively, specific food products such as potato chips and sugar-sweetened beverages which dominated the advertising landscape in this study could be initially targeted. Brand-only advertising, which made up 6.3% of all advertising in our study, also needs to be considered within the definitions of ‘healthy’ and ‘unhealthy’, given the likely substitution of companies to brand-only advertising as a way of navigating around restrictions on advertising of unhealthy foods and beverages.

From 2017, South Australia will ban alcohol advertising on buses, trains and trams [65]. This decision adopts a recommendation from a recent review of South Australian liquor licensing laws, which found that modest success in that State in increasing the age at which young people consume their first alcoholic drink could be put in jeopardy by continued exposure to alcohol advertisements on public transport and while watching sporting events [66]. The Australian Capital Territory (ACT) Government has also imposed a ban on alcohol advertising on Canberra buses which extends to advertisements for junk food, gambling, and weapons [67]. Given the failure of the ABAC code to address the placement of alcohol advertising, a similar approach could be considered for NSW, with bans extending to alcohol advertising both on and surrounding train stations. Regulatory restrictions on alcohol advertising, and on unhealthy food advertising, should be considered concurrently, given the possibility that restrictions on alcohol advertising alone may have unintended consequences by increasing unhealthy food advertisements.

## Conclusion

This is the first study to comprehensively map food and beverage advertising across a metropolitan train network in Australia, and to evaluate the nutritional quality of advertised products using the Australian Guide to Healthy Eating. The results indicate that, irrespective of season, food and beverage advertisements on the train network are overwhelmingly for unhealthy (discretionary) products, and that if bottled water is excluded, only around 1% of food advertising is for healthy (core) foods. Of particular interest is the higher proportion of unhealthy food and beverage advertising at stations in low SES areas, potentially widening the health inequalities already experienced by this group. Given that commuter train passengers are a regular and captive audience for food and beverage advertising, and close to 60% of commuters are young adults, adolescents and children, train station advertising poses a significant threat to public health. Voluntary standards adopted by the food and beverage industries are not applicable to public transport settings, and reductions in advertising of unhealthy foods and beverages in these settings are unlikely unless government introduces new ground rules. This would require adoption of a nutrient profiling system to distinguish between healthy and unhealthy foods and beverages, and either bans, targets or advertising pricing strategies to address the volume, content and placement of unhealthy food and beverage advertising.

## Additional file

Additional file 1: Survey tool. Survey tool used to record advertisement information. (PDF 277 kb)

## Abbreviations

AANA: Australian Association of National Advertisers
ABAC: Alcohol Beverages Advertising Code
ACT: Australian Capital Territory
ANOVA: Analysis of variance
ASB: Advertising Standards Bureau
CBD: Central Business District
NSW: New South Wales
OMA: Outdoor Media Association
QSRI: Quick Service Restaurant Initiative for Responsible Advertising and Marketing to Children
RCMI: Responsible Children’s Marketing Initiative
SD: Standard deviation
SES: Socioeconomic status
WHO: World Health Organisation
