# Supplementary material for: An audit of food and beverage advertising on the Sydney metropolitan train network: regulation and policy implications
Source: BMC Public Health. 2017 May 22;17:490. doi: 10.1186/s12889-017-4433-2 (PMC5440940; doi:10.1186/s12889-017-4433-2)
Supplement: Additional file 1: — Survey tool. Survey tool used to record advertisement information. (PDF 277 kb) [file 12889_2017_4433_MOESM1_ESM.pdf]

Food advertising on the Sydney metropolitan train network project

Data collector: ______________ Time: ______________________

Date: _______________________ Train station: _________________

ADVERTISEMENTS

Ad #	Product name and description	Product brand	Advertisement location	Advertisement format	Additional notes

The Boden Institute of Obesity, Nutrition, Exercise and Eating Disorders, The University of Sydney.

